# Inhibition of pathogenic bacterial carbonic anhydrases by monothiocarbamates

**DOI:** 10.1080/14756366.2023.2284119

**Published:** 2023-11-23

**Authors:** Simone Giovannuzzi, Anil Kumar Marapaka, Nader S. Abutaleb, Fabrizio Carta, Hsin-Wen Liang, Alessio Nocentini, Luigi Pisano, Mohamed N. Seleem, Daniel P. Flaherty, Claudiu T. Supuran

**Affiliations:** a Neurofarba Department, Pharmaceutical and Nutraceutical Section, University of Florence, Sesto Fiorentino (FI), Italy; b Department of Medicinal Chemistry and Molecular Pharmacology, College of Pharmacy, Purdue University, IN, USA; c Department of Biomedical Sciences and Pathobiology, Virginia-Maryland College of Veterinary Medicine, Virginia Polytechnic Institute and State University, Blacksburg, VA, USA; d Center for One Health Research, Virginia Polytechnic Institute and State University, Blacksburg, VA, USA; e Section of Dermatology, Health Sciences Department, University of Florence, Florence, Italy; f Purdue Institute for Drug Discovery, West Lafayette, IN, USA; g Purdue Institute of Inflammation, Immunology and Infectious Disease, West Lafayette, IN, USA

**Keywords:** Antibacterials, carbonic anhydrase inhibitor, monothiocarbamates, *Neisseria gonorrhoeae*, vancomycin-resistant enterococci

## Abstract

Carbonic anhydrases (CAs) from the pathogenic bacteria *Nesseria gonorrhoeae* and vancomycin-resistant enterococci (VRE) have recently been validated as antibacterial drug targets. Here we explored the inhibition of the α-CA from *N. gonorrhoeae* (α-NgCA), of α- and γ-class enzymes from *Enterococcus faecium* (α-EfCA and γ-EfCA) with a panel of aliphatic, heterocyclic and aryl-alkyl primary/secondary monothiocarbamates (MTCs). α-NgCA was inhibited *in vitro* with K_I_s ranging from 0.367 to 0.919 µM. The compounds inhibited the α-EfCA and γ-EfCA with K_I_ ranges of 0.195–0.959 µM and of 0.149–1.90 µM, respectively. Some MTCs were also investigated for their inhibitory effects on the growth of clinically-relevant *N. gonorrhoeae* and VRE strains. No inhibitory effects on the growth of VRE were noted for all MTCs, whereas one compound (**13**) inhibited the growth *N. gonorrhoeae* strains at concentrations ranging from 16 to 64 µg/mL. This suggests that compound **13** may be a potential antibacterial agent against *N. gonorrhoeae*.

## Introduction

The emergence of drug resistance to most antibacterials used clinically over the last decades^1–6^, coupled with the climate change which exacerbates this problem^7^^,^^8^, makes the finding of novel approaches to fight bacterial infections as well as agents with lower rates of resistance a crucial task for medicinal chemists and microbiologists^1^^,^^2^^,^^9^. Inhibition of bacterial carbonic anhydrases (CAs, EC 4.2.1.1) is one such approach, already proposed more than a decade ago^10^. CAs play crucial roles in bacterial metabolism and pH regulation^10–14^. Many bacterial CAs have been investigated over the last decade, and they have been validated as a bacterial target^11–14^, for several bacteria such as *Neisseria gonorrhoeae*^15^, vancomycin-resistant enterococci (VRE)^16^, *Helicobacter pylori*^17^ and *Vibrio cholerae*^18^. Most such studies have been performed with sulphonamide CA inhibitors (CAIs), one of the most investigated class of inhibitors for these enzymes. However, other classes of CAIs have recently been investigated for their inhibitory potential against bacterial CAs, such as coumarins^19^, dithiocarbamates^20^ and phenols^21^. Monothiocarbamates (MTCs) represent another class of zinc-binding CAIs^22^, which have not yet been investigated for their interaction with bacterial CAs. In this work, we report the *in vitro* inhibitory activity of a panel of MTCs against CAs of *N. gonorrhoeae* and VRE, which were reported before to be inhibited by CAIs. We do this in the search for non-sulphonamide leads which might possess antibacterial activity.

## Materials and methods

### Chemistry

MTCs **1–15** were obtained as reported earlier^22^, whereas acetazolamide **AAZ** (as standard CAI) and buffers were of >99% purity, commercially available from Sigma-Aldrich (Milan, Italy). Antibiotics were purchased commercially: Azithromycin (TCI America, OR, USA), linezolid (Chem-Impex, IL, USA), and vacomycin (GoldBio, MO, USA).

#### Enzymology and CA activity and inhibition measurements

CO_2_ hydration activity of CAs from *N. gonorrhoeae* (α-NgCA) and *E. faecium* (α-EfCA and γ-EfCA) as well as their inhibition in the presence of MTC inhibitors has been assessed by a stopped-flow method reported by Khalifah^23^. The experiments were performed at the pH of 7.4 for the α-class enzymes, and the pH of 8.3 for the γ-CA, as reported earlier^15^^,^^16^. The bacterial enzymes were recombinant proteins obtained as reported earlier by our group^15^^,^^16^.

### The minimum inhibitory concentrations (MICs) determination

The MICs were assessed using the broth microdilution method against clinically-relevant *N. gonorrhoeae* and VRE strains, as described in previous reports^15^^,^^16^^,^^24–26^. Briefly, serial dilutions of the test agent were incubated with bacteria at 37 °C either aerobically or in the presence of 5% CO_2_. MICs were determined as the lowest concentration that completely inhibited bacterial growth as observed visually.

## Results and discussion

The inhibitory effects of MTC derivatives **1–15** on three bacterial CAs isoforms, α-NgCA from N*. gonorrhoeae*, α-EfCA and γ-EfCA from *E. faecium*, were investigated (Table 1). The inhibition data of these compounds against the human offtarget isoforms hCA I and II are also given in the Table, for comparison reasons. The K_I_ values of MTCs ranged from the high nanomolar to the low micromolar ranges. The K_I_ values of MTC compounds were in the range of 0.367–0.919 µM against α-NgCA, of 0.195–0.959 µM against α-EfCA, and of 0.149–1.90 µM against γ-EfCA. In the case of α-NgCA, MTCs **1**–**4** and **6**–**11** were poorly effective inhibitors, showing K_I_ values in the high nanomolar range (0.630–0.919 µM), whereas the piperazine derivatives **5** and **13**–**15** exhibited better inhibitory effects, displaying K_I_ values of 0.367–0.417 µM. The γ-EfCA was the most effectively inhibited isoform among the bacterial CAs investigated. For instance, MTCs **7** and **12**–**15** inhibited γ-EfCA with K_I_ values of 0.149–0.357 µM, which were comparable with the K_I_ value of the standard drug **AAZ** (K_I_ = 0.322 µM). Among them, compound **14** (K_I_ = 149 nM), bearing a 4-fluorophenyl tail, resulted in 2-fold better potency than the reference drug, **AAZ**. On the other hand, the simple piperazine-tailed MTC **6** was the weakest inhibitor of γ-EfCA, with a K_I_ value in the low micromolar range (1.90 µM). In the case of α-EfCA, the piperazine derivative **11** proved to be one of the weakest inhibitors showing a K_I_ value of 0.914 µM, whereas MTCs **5**, **12**, **14** and **15** showed better inhibition constants of 290 nM, 357 nM, 195 nM and 300 nM, respectively. However, compound **14**, which was superior to **AAZ** in inhibiting the γ-EfCA, was at least 3-fold less potent than **AAZ** (K_I_ = 56 nM) in inhibiting the α-EfCA. Only the piperazine derivative **15** (NgCAα: K_I_ = 367 nM; EfCAα: K_I_ = 300 nM; EfCAγ: K_I_ = 214 nM) demonstrated a good selectivity against the discussed bacterial isozymes over the human widely expressed hCA II isoform (hCA II: K_I_ > 2 µM).

**Table 1.** Inhibition data of hCA I and II and bacterial α-NgCA, α-EfCA and γ-EfCA, using **AAZ** as a standard drug, by a stopped-flow CO_2_ hydrase assay^22^.

Name	Structure	K_I_ (µM)^a^				
		hCA I	hCA II	α-NgCA	α-EfCA	γ-EfCA
**1**	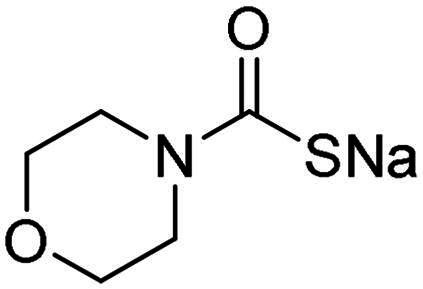	0.569	>2	0.761	0.740	1.03
**2**	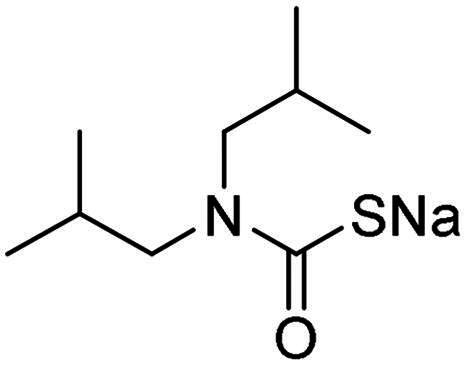	0.681	0.043	0.890	0.665	0.811
**3**	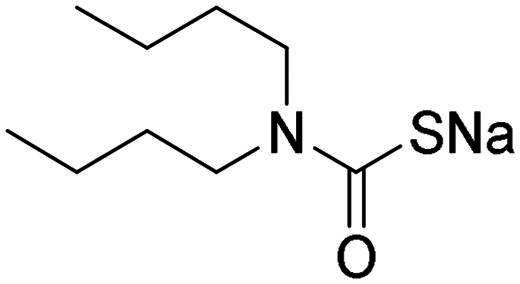	0.909	>2	0.825	0.517	0.498
**4**	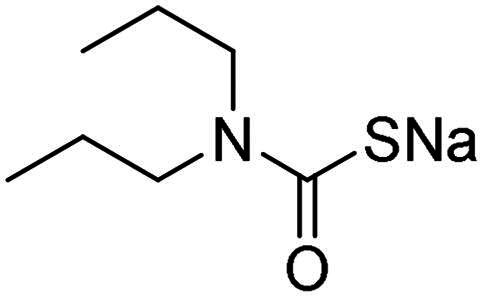	>2	0.046	0.799	0.550	0.733
**5**	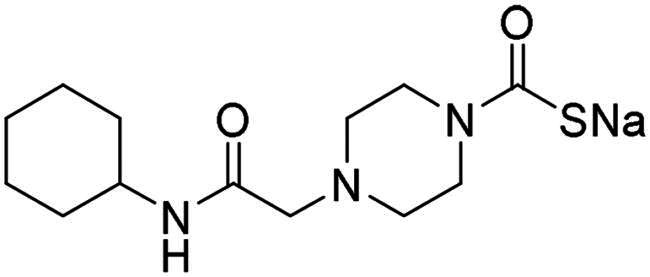	0.949	0.045	0.395	0.290	0.417
**6**	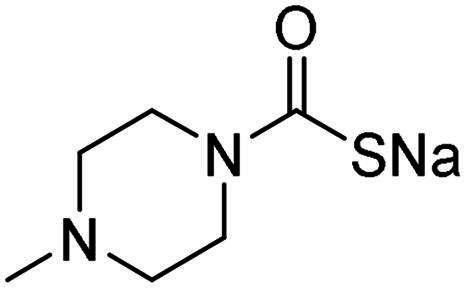	>2	0.035	0.729	0.959	1.90
**7**	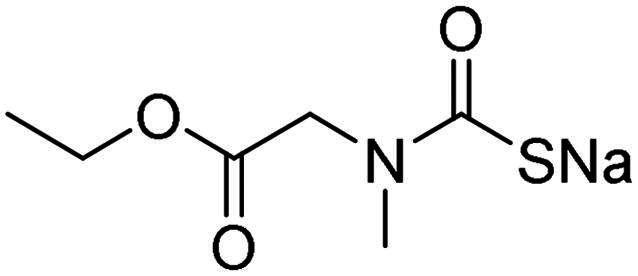	0.827	0.044	0.919	0.788	0.279
**8**	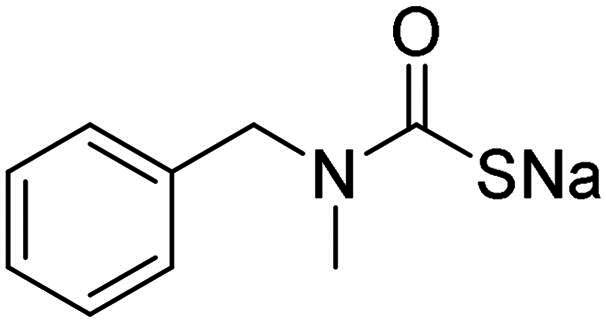	>2	>2	0.843	0.686	0.611
**9**	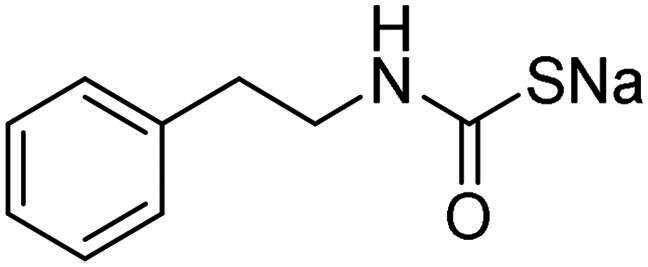	>2	0.043	0.648	0.428	0.642
**10**	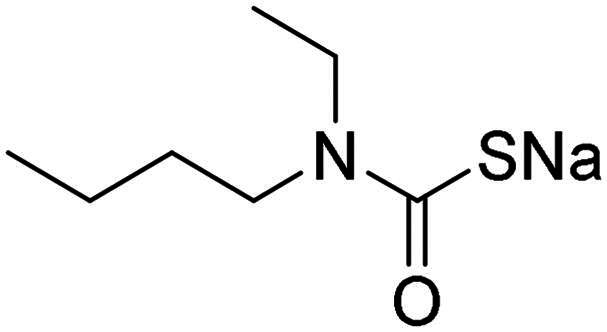	0.700	>2	0.766	0.806	0.971
**11**	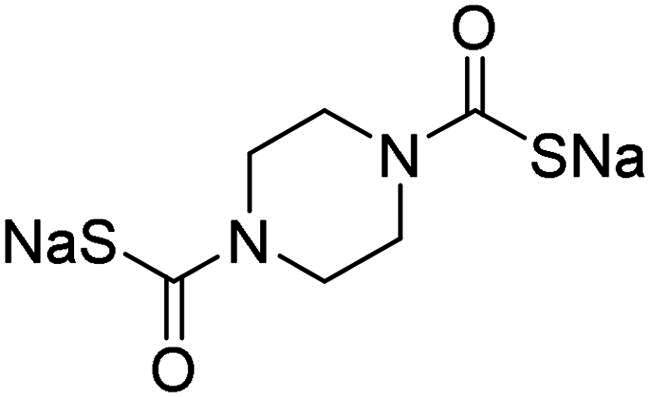	0.876	0.022	0.630	0.915	0.695
**12**	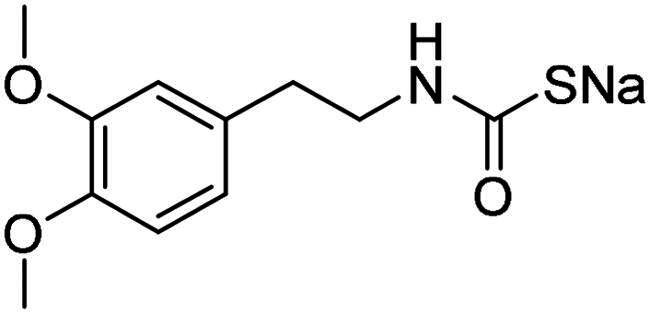	0.891	0.026	0.512	0.357	0.358
**13**	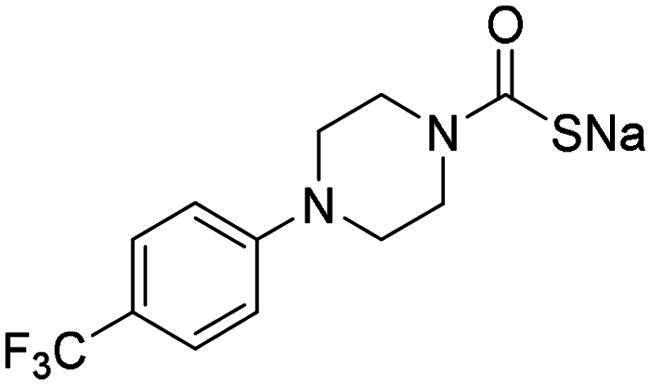	>2	0.043	0.394	0.477	0.334
**14**	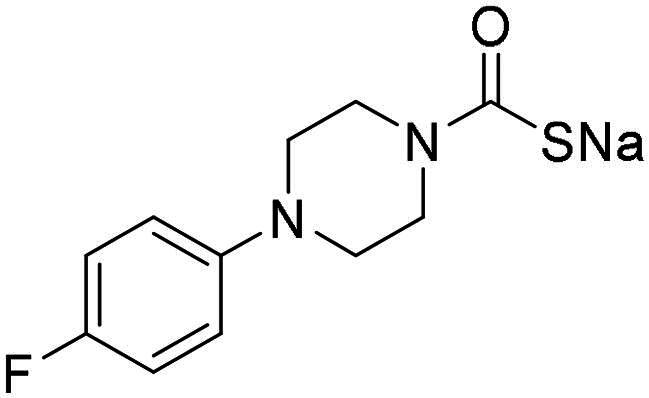	0.895	0.046	0.417	0.195	0.149
**15**	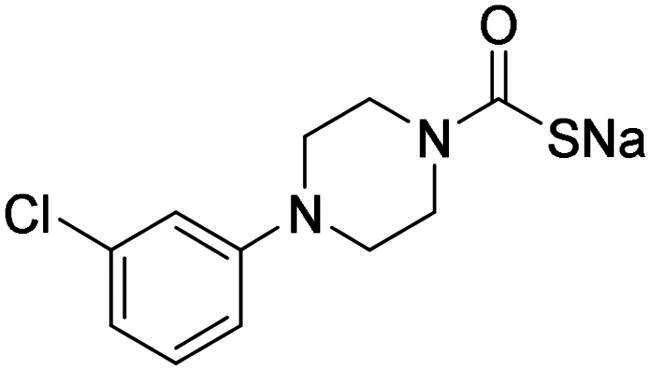	0.686	>2	0.367	0.300	0.214
**AAZ**	–	0.250	0.012	0.075	0.056	0.322

^a^Mean from 3 different assays, by a stopped-flow technique (errors were in the range of ± 5–10% of the reported values).

Next, the antibacterial activity of selected MTCs (**1**, **6**, **9**, **12** and **13**) was evaluated against a panel of multidrug-resistant strains of *N. gonorrhoeae* and VRE. Compound **13**, which has both a lipophilic trifluoromethyl-phenyl fragment and hydrophilic moieties (piperazine and monothiocarbamate functionality), displayed a modest activity against *N. gonorrhoeae* in addition to its inhibition of the α-NgCA. The compound inhibited *N. gonorrhoeae* strains with MIC values ranging between 16 and 64 µg/mL under ambient air conditions, while showing limited activity in the presence of 5% CO_2_ (Table 2). This suggests that the antigonococcal activity of **13** could be mediated by CA inhibition. Although other compounds, such as **12**, **14** and **15** showed similar *in vitro* α-NgCA inhibitory properties to **13**, only the last compound was antibacterial in vivo, presumably due to its enhanced lipophilicity due to the presence of the trifluoromethyl moiety. All the tested MTCs were inactive against the VRE strains tested (MICs >64 µg/mL) (Table 3).

**Table 2.** MICs (µg/mL) of MTCs **1, 6, 9, 12** and **13** against *Neisseria gonorrhoeae* clinical isolates.

Test agents/Control antibiotics	*Neisseria gonorrhoeae* strains					
	CDC-178		CDC-181		CDC-194	
	5% CO_2_	Ambient air	5% CO_2_	Ambient air	5% CO_2_	Ambient air
**1**	>64	>64	>64	>64	>64	>64
**6**	>64	>64	>64	64	>64	32
**9**	>64	>64	>64	>64	>64	>64
**12**	>64	>64	64	>64	>64	>64
**13**	>64	64	32	16	64	32
**AAZ**	>64	2	>64	2	>64	1
Azithromycin	2	2	>64	>64	1	0.5

**Table 3.** MICs (µg/mL) of MTCs **1, 6, 9, 12** and **13** against VRE strains.

Test agents/Control antibiotics	VRE strains					
	*E. fecalis* NR-31971		*E. fecalis* NR-31972		*E. faecium* HM-965	
	5% CO_2_	Ambient air	5% CO_2_	Ambient air	5% CO_2_	Ambient air
**1**	>64	>64	>64	>64	>64	>64
**6**	>64	>64	>64	>64	>64	>64
**9**	>64	>64	>64	>64	>64	>64
**12**	>64	>64	>64	>64	>64	>64
**13**	>64	>64	>64	>64	>64	>64
**AAZ**	>64	2	>64	2	>64	1
Linezolid	1	1	1	1	1	1
Vancomycin	>64	>64	>64	>64	>64	>64

Since the DTCs investigated earlier^20^ showed an intermediate behaviour between the ineffective MTCs and the quite effective sulphonamides^15^^,^^16^, we can speculate that the highly hydrophilic nature of the MTCs may interfere with their uptake by the bacterial cells. Thus, effective compounds targeting bacterial CAs should not only be lipophilic enough to cross the bacterial cell wall or traverse the water-filled porins, but also they should incorporate zinc-binding groups that allow a potent coordination to the active site metal ion, which is crucial both for catalysis and inhibition of these enzymes^27^.

## Conclusion

We report here the first inhibition study of bacterial CAs with MTCs, a class of CAIs that is less investigated as compared to the most well-known classes of CAIs, sulphonamides and their isosteres. CAs of *N. gonorrhoeae* (α-NgCA) and *E. faecium* (α-EfCA and γ-EfCA) were inhibited by the panel of 15 MTCs with K_I_s in the medium-high nanomolar range, with some compounds showing similar activity to the clinically used sulphonamide CAI, acetazolamide. However, the activity of these compounds in inhibiting the growth of these bacteria *in vitro* was lower as compared to the sufonamides. Thus, the MTCs seem to be less effective as bacterial CAIs, but the investigated series of compounds is too small for concluding that such compounds should not be investigated in the future.
